# *In-vitro* and *In-vivo* Hypolipidemic Activity of Spinach Roots and Flowers

**Published:** 2017

**Authors:** Mona Hafez Hetta, Abeer Sayed Moawad, Manal Abdel-Aziz Hamed, Ahmed Ismail Sabri

**Affiliations:** a *Pharmacognosy Department, Faculty of Pharmacy, Fayoum University, Fayoum, 63514, Egypt. *; b *Pharmacognosy Department, Faculty of Pharmacy, Beni-Suef University, Beni-Suef, 62514, Egypt.*; c *Therapeutic Chemistry Department, National Research Center, Dokki, Cairo, Egypt.*

**Keywords:** Spinach, Chenopodiaceae, Lipoidal matters, Flavonoid content, Hypolipidemic

## Abstract

This study was designed in order to correlate the flavonoid and lipoidal matters content of Spinach roots and flowers to their hypolipidemic potential.

The total flavonoid content was measured via complexation with aluminum chloride while determination of fatty acids methyl esters and unsaponifiable matters in both organs was performed using GC/MS. In an *in-vitro *study, the crude ethanol extracts of both organs and their different fractions were separately examined for inhibition of *β*-hydroxy-*β*-methyl glutaryl coenzyme A reductase (HMG-CoA reductase); the rate limiting enzyme of cholesterol biosynthesis.

The percentage inhibition of alcohol extracts of roots and flowers were 78.19% and 72.68% respectively when compared to the control. The crude alcohol extracts of both organs were further examined *in-vivo*. Results showed that both extracts improved the investigated parameters by variable degrees compared to fenofibrate reference drug. The root extract showed significant improvement of TC, HDL-C, LDL-C, TG and total lipids (52.75, 209.85, 21.84, 49.26 and 29.62% respectively) when compared to hypercholesterolemic rats. The histopathological picture of liver showed a noticeable amelioration after treatment with root extract. The flavonoid content was higher in flower than root (983.4 and 300.2 mg/kg respectively) while the percentage of sterols and triterpenes in roots was greater than flowers (22.47% and 17.02 % respectively).

In conclusion, the root ethanolic extract recorded more potent activity than flower as hypolipidemic agent either *in-vitro *or *in-vivo* examination which was more correlated to the sterol content than to the flavonoid content.

## Introduction

Cholesterol is an essential constituent of most biological membranes, besides acting as a precursor for the synthesis of bile acids, hormones and vitamins (1). 

High circulating cholesterol is associated with hypercholesterolemia, atherosclerosis, and stroke (2). Treatment of hyperlipidemia ideally reduces the levels of low-density lipoprotein cholesterol in the blood and attenuates the risk of the disease (3). Therefore, screening of phytochemicals as new drug candidates for managing hyperlipidemia is an encouraging trial (4). Spinach leaves (*Spinacia oleracea, *family Chenopodiaceae), has a notable flavonoid content (>1000 mg/kg) (5) comparable to other flavonoid-rich vegetables. 

The leaves are reported to contain unique flavonoid compounds including glucuronides and acylated di- and tri-glycosides of methylated and methylenedioxy derivatives of 6-oxygenated flavonols (6). Spinach phenolic compounds exhibit a wide range of biological effects including antioxidant (‎^7^), anti-inflammatory (‎^8^), antiproliferative (‎^9^) and anti-carcinogenic (10) properties; suggesting that Spinach consumption may afford protection against oxidative stress mitigated by free-radical species. Few reports dealt with the effect of Spinach leaves as hypocholesterolemic agent (11). No reports could be traced concerning the hypocholesterolemicactivity , phenolic or lipoid content of roots and flowers organs which are considered as waste products. Therefore, the aim of the present study is to compare the lipid and phenolic contents of Spinach roots and flowers and correlating their contents to the hypolipidemic activity.

## Experimental

*General experimental procedures*

Determination of saponifiable and unsaponifiable matters was performed using Agilent 6890 gas chromatography equipped with an Agilent mass spectrometric detector, with a direct capillary interface and fused silica capillary column PAS-5ms (30 m X 0.32 mm X 0.25 µm film thickness). Helium was used as a carrier gas, with flow rate 1 mL/min. The peaks were identified using Wiley and Nist 05 mass spectral data. Shimadzu UV-visible (UV-1650) spectrophotometer was used for determination of flavonoids. Petroleum ether (60-80 °C), ethyl acetate, methanol and ethanol, NaNO_2_, NaOH used were of analytical grade. AlCl_3_ and rutin (Sigma Aldrich Chemicals-Germany), fenofibrate drug (Mina Pharm., Egypt).

*Plant material*

Spinach plant has been cultivated in Beni-Suef, Egypt in March 2012. The collected plant was authenticated in Botany Department, Faculty of Science, Beni-Suef University. Voucher specimens no. BUPD 34 (a and b) was kept in Pharmacognosy Department, Faculty of Pharmacy, Beni-Suef University.

*Preparation of extractives*

The air-dried powdered organs (490 g roots and 385 g flowers) were exhaustively extracted with 70% ethanol and the solvent was evaporated under reduced pressure. A part of the residues was kept in dry clean amber glass containers in refrigerator for testing the *in-vitro *and *in-vivo *antihypercholesterolemic activity. Successive fractionation of the crude ethanol extracts of both organs, using a separating funnel, with solvents of increasing polarity; petroleum ether, EtOAc and water were carried out. The successive extractives were evaporated under reduced pressure and kept for *in-vitro* study.

*Preparation of fatty acid methyl esters (FAME) and unsaponifiable matter (USM) of Spinach roots and flowers*

One gram of the petroleum ether extract of each organ was saponified (12) by reflux with 100 mL of 10% alcoholic KOH for 8 h. USM was extracted with ether and saved for further analysis. The aqueous alkaline mother liquor was then acidified with hydrochloric acid to liberate the fatty acids then extracted with ether. The fatty acids extract was then methylated by dissolving in 50 mL methanol containing 2.5 mL sulphuric acid (13) and FAME were extracted with diethyl ether. The solvent was then distilled off and the dried residue saved for GC/MS.

*Spectrophotometric determination of the total flavonoid contents*

A weighed amount (1 g) of each of the powdered Spinach leaves (used for comparison), flowers and roots, were separately extracted with methanol (80%, 25mL), for 20 min, on an ultrasonic bath. Each extract was filtered and the filtrate was adjusted to 25 mL with methanol. The total flavonoid content was determined by adopting the aluminium chloride colorimetric assay (14). A standard curve was plotted using rutin in different dilutions of (20, 40, 60, 80, 100 µg/mL). The tested samples and standard solutions (1 mL, each) were, separately, introduced in a 10 mL volumetric flasks containing 4 mL distilled water, followed by addition of 0.3 mL 5% NaNO_2_, 0.3 mL 2% AlCl_3 _and left for 6 min before addition of 2 mL 1M NaOH. The volume was adjusted with distilled water. The absorbance was read at 510 nm against a blank solution prepared with 1mL distilled water instead of sample solution. The total flavonoid content (expressed as mg of rutin equivalent/kg dry weight), in each of the tested samples, was deduced from the standard curve.

*In-vitro hypolipidemic study*

The crude ethanol extracts and the successive fractions: petroleum ether, EtOAc and water of each organ were tested *in-vitro* for the hypolipidemic activity (15). The activity of HMG-CoA was assayed and the decrease in absorption of NADPH to NADP was measured at 340 nm. 

*In-vivo antihypercholesterolemic study*

The extracts that recorded the most potent inhibition effect of HMG-CoA was subjected to *in-vivo *hypolipidemic evaluation in hypercholesterolemic rats fed with high fat diet.

*Animals and ethics*

Male Wistar albino rats (100–120 g) were selected for this study. They were obtained from the Animal House, National Research Centre, Egypt. All animals were kept in controlled environment of air and temperature with access of water and diet. Anaesthetic procedures and handling with animals complied with the ethical guidelines of the Medical Ethical Committee of the National Research Centre in Egypt. 

*Diet*

Control groups were fed, along the experimental period (nine weeks), with standard diet (El- Kahira Co. for Oil and Soap) while hypercholesterolemic group was fed with standard diet containing 150 g lard/kg diet (16) along the nine weeks. Cholesterolemic treated groups fed with standard diet containing 150g lard/kg diet for the first nine weeks, and by starting treatments with extracts or standard drug, they fed with normal diet for 4 weeks. 

*Doses*

Administration regimens of cholesterol were five times/weeks for nine consecutive weeks (17). Extract was administrated orally at a dose 400 mg/kg body weight (18). Cholesterol was orally given at a dose of 30 mg/0.3 mL (0.7% tween)/ animal (17). Fenofibrate drug was given at a dose 50 mg/kg body weight (19).

*Experimental design*

Forty male rats were divided into five groups (eight rats each) as follows: Group 1; normal healthy control rats. Group 2; cholesterol treated rats. Groups 3 and 4; rats forced with cholesterol for nine weeks and treated with root and flower extracts respectively for 4 weeks. Group 5; rats forced with cholesterol for nine weeks and treated with fenofibrate drug for 4 weeks.

*Biochemical assays*

Blood collected from each animal by puncture the sub-lingual vein in clean and dry test tube, left 10 min to clot and centrifuged at 3000 rpm for serum separation. The separated serum was stored at -80 °C for further determinations of lipid profile and liver function tests. Serum biochemical parameters were determined using the reported methods; Cholesterol (20), high density lipoprotein- cholesterol (HDL-C) (21), low density lipoprotein-cholesterol (LDL-C) (22), triglycerides (23), total lipids (24), AST and ALT (25) and alkaline phosphatase (ALP) (26).

*Histopathological study*

Representative slices of liver tissues were fixed in 10% formalin. Paraffin-embedded sections (4-µm thick) were stained by haematoxylin and eosin (H&E) and Masson’s trichrome) Slides were seen under light microscope (27).

*Statistical analysis*

All data are expressed as mean ± SD of eight rats in each group. Statistical analysis was carried out by one-way analysis of variance (ANOVA), Costat software computer program accompanied with least significance differences (LSD) between groups at *p*<0.05. 

## Results

The total flavonoids were determined (28) and results compiled in Table 1 revealed that Spinach flowers contain higher amount of flavonoids (983.4 mg/kg) than roots (300.2 mg/kg).

Components of fatty acids, sterols and triterpenes content of roots and flowers were analyzed by GC/MS and results are presented in Tables 2 and 3.

Table 2 showed the presence of high percentage of palmitic acid (hexadecanoic acid) in the flowers (24.16%) and roots (18.73%) fatty acid components. 9, 12, 15-octadecatrienoic acid (α-linolenic, ω3) component is present in flowers as a major component (30.53%) and absent from roots. While, the 9,12-octadecadienoic acid (α-linoleic, ω6) is present in roots (0.63%) in small amount and absent in flowers.

We noticed that the percentage of hydrocarbons in flowers (19.78%) exceeds the roots (5.74%) and the major hydrocarbon in flowers was hexacosane 9-octyl (7.76%) while all hydrocarbons in roots where approximately present in equal amounts as shown in Table 3. The percentage of sterols and triterpenes in roots (22.47%) was greater than flowers (17.02 %).

The hypocholesterolemic activity of leaves has been previously studied. In this research, we studied the hypocholesterolemic activity of other organs (roots and flowers) which are considered as waste products. Table 4 includes the *in-vitro *hypolipidemic effect of plant extracts on the activity of HMG-CoA reductase; the key limiting enzyme of cholesterol biosynthesis. Results revealed that the root and flower crude alcohol extracts recorded the most inhibition percentages of this enzyme than other fractions (78.19% and 72.68% respectively) comparable to fenofibrate drug (90.75%).

Therefore, these extracts were further examined *in-vivo* as hypolipidemic agent in hypercholesterolemic rats. The hypercholesterolemic group (group 2) showed an increase in TC, TG, LDL-C and total lipids by 156.63, 60.56, 106.49 and 77.77%, respectively, while HDL-C recorded a decrease by 78.23% when compared to control group (Table 5). 

Treatment with root extract (group3) recorded significant decrease by 52.75, 21.84, 49.26 and 29.62% for TC, LDL-C, TG and total lipids, respectively as compared to hypercholesterolemic rats (group 2), while HDL-C recorded significant increase by 209.85%. Treatment with flower extract (group 4) recorded significant decrease in TC, LDL-C, TG and total lipids by 47.03, 21.03, 33.91 and 16.31%, respectively, while HDL-C recorded significant increase by 120.21% Fenofibrate drug (group 5) showed reduction by 54.75, 32.49, 51.34 and 17.24% for TC, LDL-C, TG and total lipids, while HDL-C exhibited significant increase by 143.21%. Therefore, treatment with flower extract recorded improvement by 120.70, 26.16, 33.77, 70.03 and 29.01% for TC, HDL-C, LDL-C, TG and total lipids, respectively. Root extract recorded improvement by 135.39, 45.67, 35.07, 101.72 and 52.67%, respectively. Treatment with fenofibrate drug ameliorated the lipid profile by 140.53, 31.17, 52.16, 106.02 and 30.65% for TC, HDL-C, LDL-C, TG and total lipids, respectively (Table 6).

Hypercholesterolemic rats revealed an increase in AST, ALT and ALP activities by 46.97, 57.06 and 68.83%, respectively when compared to the normal control group (Table 7).

Treatment with root extract recorded significant decrease by 30.45, 34.65 and 27.17% for AST, ALT and ALP, respectively as compared to cholesterolemic rats. Treatment with flower extract recorded significant decrease by 29.55, 27.05 and 20.00% for AST, ALT and ALP, respectively. Fenofibrate drug diminished the liver function enzymes by 27.36, 27.00 and 18.97% for AST, ALT and ALP, respectively.

Therefore, treatment with root extract improved in AST, ALT and ALP by 44.75, 54.42 and 45.88%, while flower extract exerted improvement by 43.40, 42.48 and 33.76, respectively. Fenofibrate drug showed enhancement by 40.22, 42.41 and 32.03% (Table 7). 

**Figure 1:**
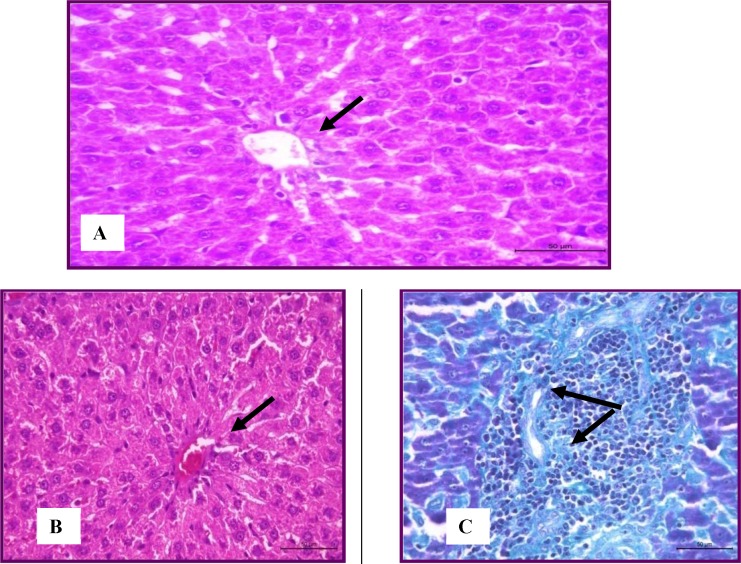
Stain (H&E) Liver of normal rats showing normal histological structure (A) and hyperlipidemic group showed mild degeneration of hepatocytes stained with (H&E) andmild leucocytic infiltration within theportal areas(Masson's trichrome) (B).

The histopathological study of the livers of control animals revealed the presence of normal histologically appearance of liver cells (Figure 1A). The hepatic lobules were normally organized and consisted from hepatic columns surrounding the central vein. The portal areas had arteries, vein and bile ducts. Hypercholesterolemic rats recorded mild degenerative changes in the hepatocytes (Figure 1B). Mild to moderate congestion the central veins and sinusoids was seen. The portal areas were mildly infiltrated with leucocytes (Figure 1C). These observations were in parallel to Zheng *et al.* (2008) (29); this was attributed to the accumulation of free radicals as a result of hypercholesterolemia.

**Figure 2.**
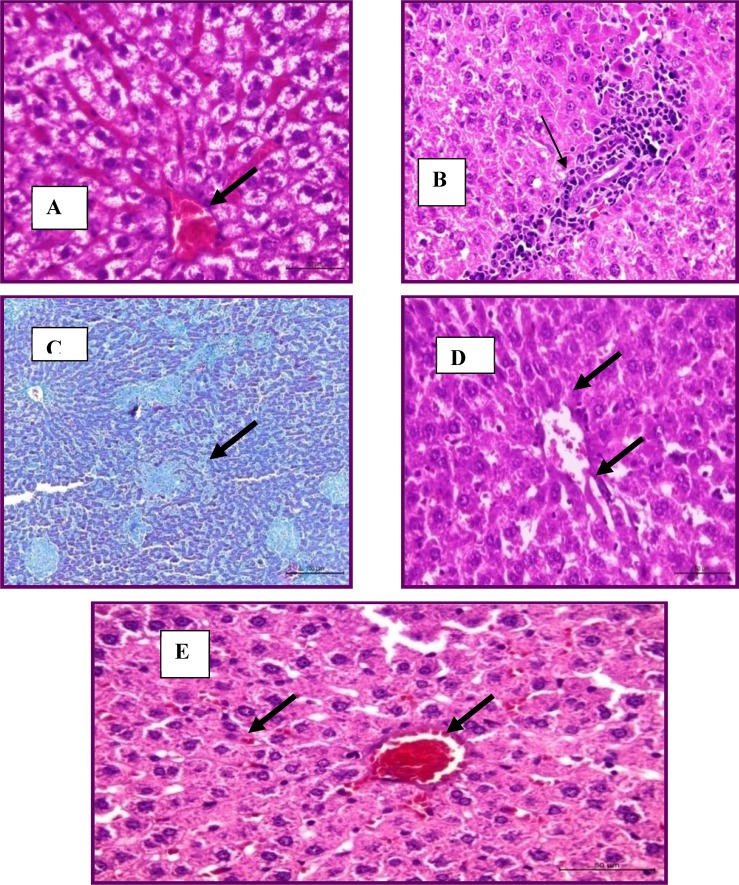
Liver treated with total flowers extract showing congested and marked dilation of hepatic sinusoids (H&E stain) (A), focal leucocytic aggregations within the portal area (H and E stain) (B) and multiple granulomas (Masson's trichrome stains) (C). Liver treated with root extract had mild degeneration (H&E stain) (D). Liver treated with drug had mild degeneration and congestion (H&E stain) (E).

The Group treated with total flowers extract showed mild degenerative changes (vacuolar degeneration and cloudy swelling) (Figure 2A). Fibrous connective tissue encapsulation was not evident in this group. Central veins were congested with marked dilation and congestion of hepatic sinusoids (Figure 2B).

Focal leucocytic aggregations could be seen within the hepatic lobules and also with the portal areas (Figure 2C). In the group treated with total root extract, the liver appeared more or less normal with the presence of very mild congestion and very mild degenerative changes (Figure 2D). No granulomas could be seen. Group treated with drug showed mild degeneration and congestion in the livers without granulomas (Figure 2E)

## Discussion

Flavonoids are secondary metabolites exerting antioxidant and chelating effects, which are beneficial to the human beings. Fatty acids composition, sterols and triterpenes of leaves of Spinach was previously studied (30), but no reports could be traced on these compounds in roots and flowers. It was reported that phytosterols showed prominent effect on cholesterol lowering ability by absorption inhibition (31), especially stigmasterol and its derivatives which are presented in roots and flowers (15.7% and 12.9% respectively) or by competing with cholesterol absorption in the gut. Stigmasta-7,16-dien-3-ol represented the major sterol in roots (9.53%) and presented also in flowers (8.65%). Both the ω6 (α-linoleic) and ω3 (α-linolenic) PUFA families are considered essential, as the human body is itself unable to synthesize them. Omega-3 fatty acids reduce blood triglyceride levels (32). 

**Table 1:** Determination of total flavonoid contents of flowers, roots and leaves of Spinach.

**Organ used**	**Flavonoids (mg/Kg)**
Flower	983.4559± 0.07
Root	300.2451± 0.12
Leaf	1537.99 ± 0.56

**Table 2:** GC/MS analysis of fatty acid methyl esters of Spinach roots and flowers.

**Roots **			**Flowers**			
**% Peak** **area**	**Compound**	**Rt**	**%Peak area **	**Compound**	**Rt**	
0.38	Tetradecanoic acid	19.55	0.46	Tetradecanoic acid	19.55	
0.43	Pentadecanoic acid	20.88	0.34	Tetradecanoic acid,12-methyl	20.51	
18.73*	Hexadecanoic acid	22.18	0.38	Pentadecanoic acid		20.89	
1.88	Heptadecanoic acid,16-methyl	24.12	0.15	Pentadecanoic acid, 14-methyl		21.70	
0.63	9,12-Octadecadienoic acid (ω6)	24.74	24.16*	Hexadecanoic acid (palmitic)	22.22	
0.74	6,9- Octadecadienoic acid	25.23	30.53*	9,12,15-Octadecadienoic acid (ω3)	24.34	
1.02	Eicosanoic acid	26.72	1.19	Nonanoic acid, 9-(o-propylphenyl)	25.01	
0.55	Octadecanoic acid,11-methyl	28.39	2.59	10,13- octadecadienoic acid	25.25	
			0.38	4,8,12,16 tetramethyl heptadecan -4-olide	27.06	

* major compounds

**Table 3:** GC/MS analysis of unsaponifiable matter of roots and flowers of Spinach.

**Roots **			**Flowers**		
**%Peak**	**Compound**	**Rt**	**% Peak**	**Compound**	**Rt**
0.63	Cycloeicosane	26.32	1.76	1-Tricosene	28.38
0.58	1-Nonadecene	30.32	1.20	Heptacosane	30.31
0.98	Octacosane	35.12	7.76*	Hexacosane, 9-octyl	31.00
0.29	Cholestan-3-one, 4,4-dimethyl	38.59	1.24	Nonacosane	32.34
0.84	Ergost -7-en-3-ol	39.03	2.15	Eicosane, 10-heptyl-10-octyl	33.24
0.64	Ergosta-8,24(28)-dien-3-ol, 4,14-dimethyl	39.32	1.96	Nonacosan-10-ol	34.61
9.53*	Stigmasta-7,16-dien-3-ol	40.02	1.03	1-Nonadecene	36.57
1.66	Stigmastan-3-ol	40.24	8.65*	Stigmasta-7,16-dien-3-ol	39.78
2.84	Lanost-24-en-3-ol	40.92	2.65	Lanosta-8,24-dien-3-ol	40.75
4.51	Stigmasta-7-en-3-ol	41.48	4.24	Stigmasta-7-en-3-ol	41.28
1.71	Pregn-15-en-20-one	41.91	1.48	α-amyrin	41.89
2.16	Lanosta-8,24-dien-3-ol	42.24	2.68	9,19-Cycloanostan-3-ol, 24-methyl	43.54
1.84	9,19-Cycloanostan-3-ol, 24-methyl	43.67			

* major compounds

**Table 4:** *In-vitro *hypolipidemic activity of Spinach roots and flowers

**Sample**	**Enzyme activity** * **(µmole/mg dried extract)**	**Inhibition ** **(%)**
Control	16.33±0.33	----
(Fenofibrate; 100µg)	1.51±0.23	90.75%
**Root**		
Total 70% EtOH	3.56±0.21	78.19%
Petroleum ether	4.41±0.29	72.99%
EtOAc	5.49±0.13	66.38%
H_2_O	5.60 ± 0.22	65.57%
**Flower**		
Total 70% EtOH	4.67±0.10	72.68%
Petroleum ether	6.42±0.09	60.68%
EtOAc	7.36±0.06	54.92%
H_2_O	6.22 ±0.19	61.91%

Data are mean± SD of three readings.

% of inhibition=Control value- test value x 100 /Control value.

* *β*-hydroxy-*β* glutaryl CoA- reductase)

**Table 5:** Effect of treatment with roots and flowers extracts of Spinach on lipid profile of hypercholesterolemic rats.

**Parameter**	**Group**				
	**1**	**2**	**3**	**4**	**5**
TC	113.00±9.08^d^ ---	290.00±19.68^a^ (+156.63)	137.00±7.58^c^ [-52.75]	153.60±7.89^b^ [-47.03]	131.20±5.01^c^ [-54.75]
HDL-C	62.94±3.56^a^ ---	13.70±1.66^d^ (-78.23)	42.45±4.28^b^ [+209.85]	30.17±7.08^c^ [+120.21]	33.32±5.64^c^ [+143.21]
LDL-C	69.93±13.24^b^ ---	112.28±5.60^a^ (+60.56)	87.75±15.07^b^ [-21.84]	88.66±21.86^b^ [-21.03]	75.80±15.41^b^ [-32.49]
TG	130.57±3.68^c^ ---	269.62±25.24^a^ (+106.49)	136.80±3.08^c^ [-49.26]	178.17±9.20^b^ [-33.91]	131.18±12.34^c^ [-51.34]
Total lipids	4.86±0.58^d^ ----	8.64±0.41^a^ (+77.77)	6.08±0.22^c^ [-29.62]	7.23±0.52^b^ [-16.31]	7.15±0.29^b^ [-17.24]

●Data are means ± SD of eight rats in each group.

●Data are expressed as mg/dL. Total lipids are expressed as g/dL.

●Unshared superscript letters between groups are the significance values at *p*< 0.0001.

●Values between brackets are percentage change over control group.

●Values between parentheses are percentage change over cholesterolemic group.

●Statistical analysis is carried out by one-way analysis of variance (ANOVA) – Costatsoftware computer program accompanied with LSD between groups at *p<*0.05.

●1: control group; 2: cholesterol untreated group; 3: group treated with root extract; 4: group treated with flower extract; 5: fenofibrate-treated group.

**Table 6:** Effect of treatment with roots and flowers extracts of Spinach on liver function enzymes of hypercholesterolemic rats

**Parameter**	**Group**				
	**1**	**2**	**3**	**4**	**5**
AST	37.02±2.27^b^ ---	54.41±4.31^a^ (+46.97)	37.84±5.69^b^ [-30.45]	38.33±3.23^b^ [-29.55]	39.52±1.94^b^ [-27.36]
ALT	42.06±4.75^c^ ---	66.06±3.11^a^ (+57.06)	43.17±3.11^c^ [-34.65]	48.19±3.19^b^ [-27.05]	48.22±2.67^b^ [-27.00]
ALP	46.20±1.92^d^ ---	78.00±1.22^a^ (+68.83)	56.80±1.30^c^ [-27.17]	62.40±2.30^b^ [-20.00]	63.20±1.92^b^ [-18.97]

●Data are means ± SD of eight rats in each group, Data are expressed as Unit/L.

●Unshared superscript letters between groups are the significance values at *p*< 0.0001.

●Values between brackets are percentage change over control group.

●Values between parentheses are percentage change over cholesterolemic group.

●Statistical analysis is carried out by one-way analysis of variance (ANOVA) – Costat software computer program accompanied with LSD between groups at *p*<0.05.

●1: control group; 2: cholesterol untreated group; 3: group treated with root extract; 4: group treated with flower extract; 5: fenofibrate- treated group.

**Table 7:** Percentage of improvement of lipid profile and liver function after treatment with crude alcohol extracts of roots and flowers

**Parameters**	**Group**		
	**3**	**4**	**5**
TC	135.39%	120.70%	140.53%
HDL-C	45.67%	26.16%	31.17%
LDL-C	35.07%	33.77%	52.16%
TG	101.72%	70.03%	106.02%
Total lipids	52.67%	29.01%	30.65%
AST	44.75	43.40	40.22
ALT	54.42	42.48	42.41
ALP	45.88	33.76	32.03

●Values are % of improvement over control group.

●of improvement %= [(mean treated - mean cholesterolemic)/mean control]× 100.

●**3** group treated with root extract; **4**: group treated with flower extract; **5**: fenofibrate-treated group.

Adaramoye *et al.* (2008) (17) attributed the reduction of cholesterol and triglycerides levels after treatment to the reduction of hepatic triglyceride biosynthesis and redistribution of cholesterol among the lipoprotein molecules. In addition, reduction of total cholesterol by plant extract was associated with a decrease of its LDL fraction, which is the target of several hypolipidemic drugs. Therefore, cholesterol-lowering activity of plant extract could result from rapid catabolism of LDL-C through its hepatic receptors for final elimination in the form of bile acids. Tsutomu *et al.* (1993) (33) added that the possible mechanisms of cholesterol reduction includes the increase of faecal cholesterol and fatty acids excretion; increase excretion of faecal bile acids and neutral steroids and the presence of high fibre food that replace fat and cholesterol-containing foods (34). Treatment with fenofibrate significantly decreased triglycerides, while the other lipid profiles were not significantly changed. These findings agree with the mechanism of action of fibrates, where LDL-C lowering activity is not marked but triglycerides decreasing effect is spectacular, through stimulation of the lipoprotein lipase gene, which enhances catabolism of VLDL, synthesis of fatty acids and reduces VLDL secretion (35).

Treatment with root alcohol extract recorded significant decrease by 30.45, 34.65 and 27.17% for AST, ALT and ALP, respectively as compared to cholesterolemic rats. Treatment with flower extract recorded significant decrease by 29.55, 27.05 and 20.00% for AST, ALT and ALP, respectively. Awad *et al.* (2012) (36) attributed the increase in enzymes activity to the hypercholesterolemic state that led to elevation of free radicals and lipid peroxidation process, affected the permeability of hepatocyte membranes and enzyme leakage into the circulation.

The improvement of the histopathological picture of liver was attributed to the effect of the biologically active compounds present in the extract. As previously reported (37); Spinach dietary fibre, in addition to its potent triglyceride and total cholesterol-lowering effect, has antioxidant effect for reducing lipid peroxidation in plasma and tissues, and enhancing the antioxidant enzymes in rats fed high-cholesterol diet.

## Conclusion

It could be concluded that the total crude ethanol extract of Spinach roots recorded more potent effect than the flowers as hypolipidemic agent. These organs could be of great merit commercially. Treatment with Spinach plant extracts led to significant elevation of serum HDL-C, indicating its promising protective role against cardiovascular disease. The effect could be related to the synergistic effect of the chemical components; fatty acids, sterols and flavonoids. 
